# Transcriptome Analysis in Air–Liquid Interface Porcine Respiratory Epithelial Cell Cultures Reveals That the Betacoronavirus Porcine Encephalomyelitis Hemagglutinating Virus Induces a Robust Interferon Response to Infection

**DOI:** 10.3390/v16060939

**Published:** 2024-06-11

**Authors:** Kaitlyn M. Sarlo Davila, Rahul K. Nelli, Juan C. Mora-Díaz, Yongming Sang, Laura C. Miller, Luis G. Giménez-Lirola

**Affiliations:** 1 Infectious Bacterial Disease Research Unit, National Animal Disease Center, United States Department of Agriculture, Agricultural Research Service, Ames, IA 50010, USA; kaitlyn.sarlodavila@usda.gov; 2 Department of Veterinary Diagnostic and Production Animal Medicine, College of Veterinary Medicine, Iowa State University, Ames, IA 50011, USA; rknelli@iastate.edu (R.K.N.); juanmora@iastate.edu (J.C.M.-D.); 3 Department of Agricultural and Environmental Sciences, College of Agriculture, Tennessee State University, Nashville, TN 37209, USA; ysang@tnstate.edu; 4 Virus and Prion Research Unit, National Animal Disease Center, United States Department of Agriculture, Agricultural Research Service, Ames, IA 50010, USA; 5 Department of Diagnostic Medicine/Pathobiology, College of Veterinary Medicine, Kansas State University, Manhattan, KS 66506, USA

**Keywords:** ALI-PREC, organoid, PHEV, betacoronavirus, transcriptomics, chemokines, mucociliary

## Abstract

Porcine hemagglutinating encephalomyelitis virus (PHEV) replicates in the upper respiratory tract and tonsils of pigs. Using an air–liquid interface porcine respiratory epithelial cells (ALI-PRECs) culture system, we demonstrated that PHEV disrupts respiratory epithelia homeostasis by impairing ciliary function and inducing antiviral, pro-inflammatory cytokine, and chemokine responses. This study explores the mechanisms driving early innate immune responses during PHEV infection through host transcriptome analysis. Total RNA was collected from ALI-PRECs at 24, 36, and 48 h post inoculation (hpi). RNA-seq analysis was performed using an Illumina Hiseq 600 to generate 100 bp paired-end reads. Differential gene expression was analyzed using DeSeq2. PHEV replicated actively in ALI-PRECs, causing cytopathic changes and progressive mucociliary disruption. Transcriptome analysis revealed downregulation of cilia-associated genes such as *CILK1*, *DNAH11*, *LRRC-23*, *-49*, and *-51*, and acidic sialomucin *CD164L2*. PHEV also activated antiviral signaling pathways, significantly increasing the expression of interferon-stimulated genes (*RSAD2*, *MX1*, *IFIT*, and *ISG15*) and chemokine genes (*CCL5* and *CXCL10*), highlighting inflammatory regulation. This study contributes to elucidating the molecular mechanisms of the innate immune response to PHEV infection of the airway epithelium, emphasizing the critical roles of the mucociliary, interferon, and chemokine responses.

## 1. Introduction

Porcine hemagglutinating encephalomyelitis virus (PHEV) is a highly contagious swine pathogen associated with vomiting and wasting disease (VWD), encephalomyelitis, and/or respiratory disease [1,2,3]. PHEV is an enveloped single-stranded, positive-sense RNA coronavirus in the species *Betacoronavirus 1* in the family *Coronaviridae* [4]. The disease was described for the first time in 1957 in nursing pigs in Ontario, Canada [5]; however, the virus was not isolated until 1962 during an encephalomyelitis outbreak in neonatal pigs [6]. Clinical manifestations of PHEV are restricted to piglets younger than 4 weeks of age, including VWD and encephalomyelitis [2,3,7]. The neurotropism of PHEV was also demonstrated in mice and rats but only under experimental conditions [8,9]. Although PHEV can infect any age, older pigs develop self-limiting or subclinical infections [10,11].

Primary replication of PHEV in pigs occurs in the respiratory tract, followed by infection of peripheral nerves and subsequent spread to the central nervous system (CNS) [12]. In a previous study we demonstrated in vivo and ex vivo that PHEV strain 67 N [13] replicates in the upper respiratory tract and tonsils using both a refined cesarean-derived, colostrum-deprived (CDCD) neonatal pig model and an organotypic air–liquid interface (ALI) porcine respiratory epithelial cell culture (PREC) system derived from CDCD neonatal pig tracheas [14].

Under ALI culture conditions, primary porcine cells derived from the respiratory tract are differentiated into a pseudostratified respiratory epithelium consisting of ciliated cells, goblet/secretory cells, and basal cells [15,16,17]. The ALI-PREC culture model was demonstrated to be sufficiently complex to resemble the biological and physiological properties of the respiratory tract’s epithelial lining and constitutes a viable alternative infection model to investigate the molecular mechanisms of the innate immune response and pathogenesis of respiratory pathogens [18,19].

Our previous studies on innate immune response to PHEV (67 N strain) using an ALI-PREC culture system revealed that upon PHEV infection of porcine respiratory cells, pattern recognition receptors (PRRs), such as Toll-like receptor 3 (TLR3), TLR7, retinoic acid-inducible gene (RIG)-I, and MyD88 played a crucial role in the transcriptional upregulation of *IFN-λ* 1 gene, which in turn upregulated the transcription factor STAT1, and therefore induced the expression of the antiviral IFN-stimulated genes *OAS1* and *Mx1* [20]. In addition, PHEV infection increased the secretion of IL-8 in grower and neonatal pigs, as well as in the subnatant of ALI-PREC cultures infected with the virus [14,20]. IL-8 plays a key role in the recruitment and subsequent infiltration of T-lymphocytes and macrophages [21,22].

Understanding the immune pathways responding to PHEV replication is essential for developing effective strategies to prevent and control diseases associated with this pathogen. The objective of this study is to further our understanding of the molecular mechanisms of the pathogenesis and innate immune response towards PHEV infection of ALI-PREC culture via transcriptomic analysis.

## 2. Materials and Methods

### 2.1. Culturing ALI-PRECs

Isolation and culture of PRECs were performed as previously described [20]. Tracheal samples were washed with phosphate-buffered saline (PBS) supplemented with 100 IU/mL of penicillin/100 µg/mL of streptomycin (Pen–Strep; Thermo Fisher Scientific Inc., Waltham, MA, USA) and 1.25 µg/mL of amphotericin B (AmpB; Thermo Fisher Scientific). Tissue digestion was performed in calcium and magnesium-free Minimum Essential Medium (MEM; in-house), supplemented with 1.4 mg/mL pronase (Millipore-Sigma, Burlington, MA, USA), 0.1 mg/mL DNase (Millipore-Sigma), 100 µg/mL Primocin (Invivogen, San Diego, CA, USA). The dissociated cells (PRECs) were frozen at −80 °C in an LHC^®^ basal medium (Thermo Fisher Scientific) containing 30% heat-inactivated fetal bovine serum (EqualFetal; Atlas Biologicals, Fort Collins, CO, USA) and 10% dimethyl sulfoxide (DMSO; Millipore-Sigma).

As described previously [20], isolated PRECs were seeded at a density of ~20,000 cells/mm^2^ on 24-well ThinCert cell culture inserts or “transwell-inserts” [Polyethylene terephthalate (PET); transparent; pore size 0.4 μm; pore density 2 × 10^6^ cm^2^; Greiner Bio-One North America Inc., Monroe, NC, USA) coated with collagen from the human placenta-Bornstein and Traub Type IV (Millipore-Sigma). Cells were cultured in a growth medium containing DMEM/F12 (Thermo Fisher Scientific) supplemented with 1400 nM hydrocortisone (Acros Organics, Fair Lawn, NJ, USA), 2700 nM epinephrine (Acros Organics), 100 nM retinoic acid (Acros Organics), 9.7 nM 3,3′,5-Triiodo-L-thyronine (Cayman Chemicals, Ann Arbor, MI, USA), 0.5 ng/mL murine epidermal growth factor (EGF; PeproTech US, Rocky Hill, NJ, USA), 1X insulin-selenium-transferrin (Thermo Fisher Scientific), 1X 4-(2-hydroxyethyl)-1-piperazineethanesulfonic acid (HEPES; Thermo Fisher Scientific), 2% Ultroser-G (Pall France, Cergy, France), Pen–Strep, and AmpB. The growth medium was replaced every 2–3 days until PRECs were completely differentiated into ALI-PRECs with no visible medium seepage, a shiny glaze that resembled mucus and cilia development.

### 2.2. PHEV In Vitro Culture and Propagation

PHEV 67 N or “Mengeling strain” was obtained from the National Veterinary Services Laboratories [NVSL, United States Department of Agriculture (USDA), Ames, IA, USA] and was propagated in swine kidney primary (SKP) cells (NVSL), as described previously [14]. The virus harvested in this study was titrated by hemagglutination assay (HA) and stored at −80 °C.

### 2.3. PHEV Infection in ALI-PRECs

Approximately 27–30 days old, completely differentiated ALI-PRECs from two independent control cesarean-derived, colostrum-deprived (CDCD) pigs (i.e., 2 biological and 3 technical replicates) were used for PHEV infection studies as previously described [20]. The number of replicates (*n* = 6 at each time point and treatment) ensures the study is statistically robust. On transwells inserts, a volume of 250 µL of 1:1 dilution of PHEV 67 N (1:128 HA titer) and infection medium containing DMEM/F12 supplemented with 2% Ultroser G, 1X MEM non-essential amino acids, 1X HEPES, Pen–Strep, and 2 µg/mL N-tosyl-L-phenylalanine chloromethyl ketone (TPCK) treated trypsin (Millipore-Sigma) was used. Infection medium without the virus was used for mock inoculations and platewells subnatants of both treatments to control for any non-specific effects. After 6 h at 37 °C and 5% CO_2,_ the inoculum was removed, the cell cultures (i.e., transwell and platewell) were washed once with DMEM/F12, and fresh infection medium was added into the platewells only, exposing the transwell surface to air. Subsequently, plates were incubated for 24, 36, and 48 h post-inoculation (hpi) at 37 °C with 5% CO_2_. ALI-PRECs cultures were monitored daily under the microscope for cytopathic changes.

### 2.4. PHEV RT-qPCR

Viral RNA extractions were performed using the E.Z.N.A.^®^ Viral R.N.A. Kit (Omega Bio-tek, Inc., Norcross, GA, USA) and vacuum manifold (QIAGEN, Germantown, MD, USA) method following the manufacturer’s instructions. A quantitative PHEV N gene-based RT-PCR (RT-qPCR) developed by Tetracore (Tetracore, Inc., Rockville, MD, USA) and Iowa State University [11] was used to confirm and quantify PHEV infection in vivo (CDCD pigs) and ex vivo (ALI-PRECs cultures). Each RT-qPCR reaction (25 μL final reaction volume) was set up by combining 19 μL of PHEV RT-qPCR master mix and 1 μL of the enzyme blend (reverse transcriptase and RNase inhibitor). An internal control (IC) was used as an extraction control, with 6 μL of the IC added to the lysis buffer. Then 5 μL of the extracted sample RNA with IC was added to Master Mix. All RT-qPCR reactions were performed in duplicate, with a negative extraction control (NEC), positive extraction control (PEC), and a “no template” control (NTC) included in each run. RT-qPCR reactions were run on a Rotor-Gene Q (QIAGEN) with cycling conditions; 48 °C for 15 min and 95 °C for 2 min holding; 45 cycles; 95 °C for 10 s denaturation; and 60 °C for 40 s amplification. The RT-qPCR results were analyzed using Rotor-Gene Q Pure Detection software (v 2.3.1). Samples with a threshold cycle (Ct) above 40 were considered negative.

### 2.5. Total RNA Isolation, Reverse Transcription, and Transcriptional Analysis

According to the manufacturer’s protocol, total RNA from ALI-PRECs was isolated for transcriptomic analysis using TRizol reagent (Thermo Fisher Scientific), chloroform phase separation, and commercially available RNeasy Plus Mini Kit (QIAGEN). Eluted RNA was quantified using a NanoDrop one microvolume UV-Vis spectrophotometer (Thermo Fisher Scientific), and samples with A260/280 between 1.96 and 2.05 were used for reverse transcription using the qScript XLT cDNA SuperMix Kit (Quantabio, Beverly, MA, USA). Furthermore, RNA quality was assessed with the Agilent bioanalyzer. The total RNA from each sample was used for RNA-Seq libraries, and indexed libraries for individual samples were pooled and sequenced utilizing the Illumina Hiseq 6000 to generate 100 base paired-end reads. Both library preparation and sequencing were performed at the Iowa State University Genomics Center. An average of 14,330,993.17 reads were generated per sample.

All qPCR reactions were performed using 1× PowerUp SYBR Green Master Mix (Thermo Fisher Scientific), 500 nM of swine-specific primers as described previously [20], 1.5 ng of total RNA converted to cDNA on Applied Biosystems 7500 Fast real-time system (Applied Biosystems, Foster City, CA, USA) thermocycler. The qPCR results were analyzed using 7500 Software v2.3 (Applied Biosystems) and exported to Microsoft Excel (Microsoft Corporation, Redmond, WA, USA). Amplification efficiencies beyond the range (1.5–2.2), samples with multiple melting peaks and above 40 Ct, were discarded. Ct values were subsequently analyzed on qBase+ gene expression analysis software (Biogazelle, Zwijnaarde, Belgium), which calculates the stability of endogenous control genes and provides a value called M-value. Out of seven endogenous control genes (*ACTB*, *B2M*, *EIF3K*, *GAPDH*, *PPIA*, *RPL10*, *PCNA*) used, three genes (*EIF3K*, *PPIA*, and *RPL10*) were identified as the best endogenous controls based on their low M-value. Hence, gene expression data were normalized using the geometric mean of these three genes [23]. Relative quantitation analysis was performed using the ΔΔCt method as described previously [24].

### 2.6. Differential Gene Expression Analysis

Tools at galaxy.scinet.usda.gov were utilized to analyze the sequenced reads and examine raw read data and counts. MultiQC was used to combine and visualize the FastQC results for all samples. The phred score and counts for each sample were performed Trimmomatic (version 0.38.1) [25] was used to remove adapters and reads with a phred score below 20 using a sliding window averaged across four bases prior to alignment on the Sscrofa 11.1 genome assembly with HiSat2 (version2.1.0) [26,27] utilizing default parameters for paired-end reads (FR option for orientation). Raw counts were generated with the default parameters (without count split and non-split alignments or fragment counts) of FeatureCounts and the NCBI Sscrofa11.1 version 2.2 GTF file. Differential gene expression (DEG) was performed using DeSeq2 (version 2.11.40.6) [28], utilizing a parametric fit type and poscounts to account for genes with zero counts. DEG analysis was based on the model treatment + hpi + treatment:hpi + E. Statistically significant genes were reported for the interaction effect of treatment:hpi. Genes were declared statistically significant at the Benjamini–Hochberg false discovery rate (FDR) adjusted *p* < 0.15.

### 2.7. Gene Ontology and Network Analysis

Gene Ontology (GO) term enrichment and clustering analysis were performed on the DEGs from each timepoint using the ClueGo Plug-in in Cytoscape 3.9.1 software [29]. GO term enrichment was calculated with a hypergeometric test. Functional grouping was based on the kappa score. Terms were declared as connected with a kappa score greater than 0.4. Redundant groups with greater than 50% overlap were merged. Mapped genes represent at least 4% of the total associated genes per term.

### 2.8. Pathway Analysis

Ingenuity Pathway Analysis (IPA) by Qiagen was used to determine the top canonical pathways associated with the DEGs data from ALI-PRECs at each time point. Significance values for the canonical pathways were calculated using the right-tailed Fisher’s Exact Test. The ratio is calculated as the number of significantly differentially expressed molecules in each pathway divided by the total number of molecules in that pathway. The IPA z-score values were used to predict the direction of change in each pathway, indicating the predicted activation or inhibition. Positive z-scores indicate predicted activation, while negative z-scores indicate predicted inhibition. Z-scores were only calculated for pathways including at least four significantly differentially expressed genes. The differential gene expression was visualized for each time point for a significant pathway. Based on significant z-score values, the molecule activity predictor (MAP) tool was used to predict upstream and downstream effects of activation and inhibition based on these known changes in gene expression.

## 3. Results

### 3.1. PHEV Replicates in ALI-PREC Cultures

**Figure 1:**
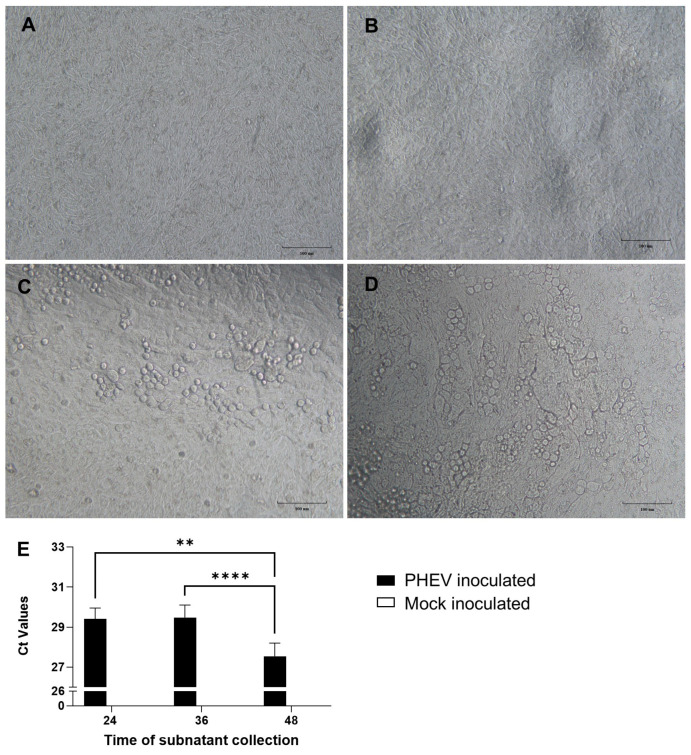
Air–liquid interface porcine respiratory epithelial cells (ALI-PREC) susceptibility toward porcine hemagglutinating encephalomyelitis virus (PHEV) infection. (**A**–**D**) Completely differentiated ALI-PRECs (day 30) treated with infection medium only, i.e., without virus (mock-inoculated) (**A**), (**B**) with HA (titer of 128) of PHEV 67 N for 24 h post-inoculation (hpi), (**C**) 36 hpi, and (**D**) 48 hpi. Bar, 100 μm. Representative images from two biological and three technical replicates. (**E**) Detection of PHEV nucleocapsid gene using reverse transcription-qPCR. RNA from the subnatants collected from ALI-PRECs treated with PHEV was analyzed using RT-qPCR developed by ISU and Tetracore. Collection time is shown in hours. A sample volume of 5 μL of extracted sample RNA along with internal control was added to the qPCR master mix. All qPCRs were performed with a negative extraction control (NEC), a positive extraction control (PEC), and a no-template control (NTC) included in each run. Samples from two biological replicates and three technical replicates. Statistical analysis was performed using Fisher’s LSD multiple-comparison test (GraphPad Prism 9.0.1). **, *p* value  <  0.01, and ****, *p* value  <  0.0001.

Following PHEV inoculation, ALI-PRECs appeared normal and showed active ciliary movement in both mock- and virus-inoculated cells up to 24 h hpi. The virus-inoculated cells started showing cytopathic changes such as ciliary destruction, cytoplasmic stranding, vacuolation, rounding of cells, clusters of rounded cells, cell shrinkage, and detachment of cells around 36 hpi and increased over 48 hpi compared to mock-inoculated cells (Figure 1 A–D). Once the integrity of ALI-PRECs was breached, the virus started to shed into the bottom subnatants of the plate well medium, as evidenced by the RT-qPCR results (Figure 1E).

### 3.2. PHEV-Induced Robust Transcriptional Regulation in ALI-PRECs

**Figure 2:**
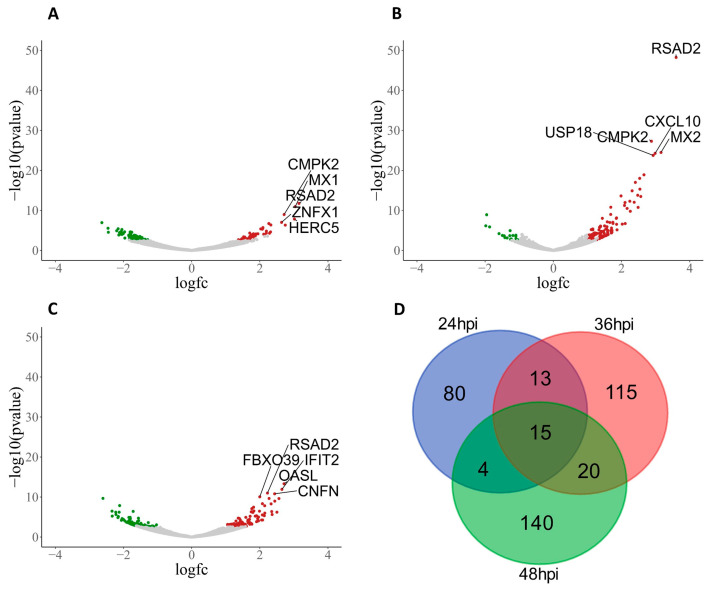
PHEV-induced DEG in ALI-PRECs at 24, 36, and 48 h post-infection (hpi). Volcano plots show differential gene expression at 24 hpi (**A**), 36 hpi (**B**), and 48 hpi (**C**). Significant genes (Benjamini–Hochberg FDR *p* < 0.15) downregulated are shown in green, while upregulated genes are shown in red. Genes that are not significantly differentially expressed are shown in grey. The number of DEGs shared between time points is plotted in the Venn diagram (**D**).

Following PHEV infection, the significant (FDR < 0.15) number of DEGs increased throughout infection with 112, 163, and 179 DEGs at 24, 36, and 48 hpi, respectively (Tables S1–S3), and corresponding volcano plots (Figure 2A–C). At 24 hpi, 50 DEGs were upregulated, and 62 DEGs were downregulated in the infected samples compared to the controls (Table S1). A statistically significantly greater number of DEGs upregulated by 36 hpi, with 132 DEGs up and only 31 DEGs down compared to the controls (Table S2). Expression profiles were more balanced at 48 hpi with 97 upregulated DEGs and 82 downregulated DEGs (Table S3). A total of 15 DEGs (*RSAD2*, *CMPK2*, *HERC5*, *HERC6*, *GBP1*, *OAS2*, *OASL*, *MX1*, *MX2*, *ISG15*, *IFIT1*, *ENSSSCG00000017754*, *EPSTI1*, *UBE2L6*, and *PSMB9*) were shared across all three time points (Figure 2D). Interestingly, the top three significantly (*p* value  <  0.000001) upregulated genes at 24 (*RSAD2*, *CMPK2*, and *MX1*), 36 (*RSAD2*, *CMPK2*, and *MX2*), and 48 (*RSAD2*, *OASL*, and *IFIT2*) hpi belong to antiviral activity, with a fold change >2.5 (Tables S1–S3).

### 3.3. Network Visualization and Analysis of DEGs in ALI-PRECs following PHEV Infection

**Figure 3:**
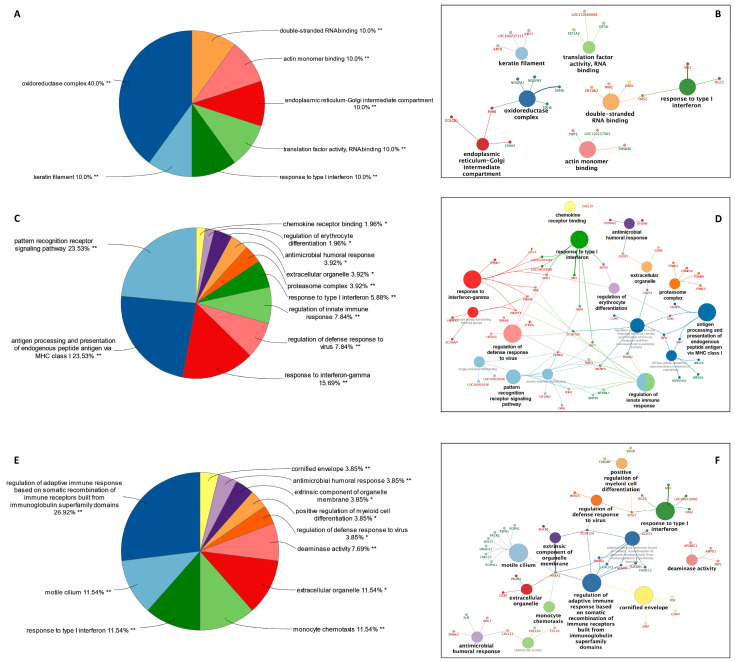
Network visualization of DEG in ALI-PRECs at 24, 36, and 48 h post-infection (hpi) with PHEV. Percentage GO terms per group (grouped by kappa score) in PHEV-infected ALI-PRECs at (**A**) 24 hpi, (**C**) 36 hpi, (**E**) 48 hpi. Network of functionally grouped significant gene ontology terms at (**B**) 24 hpi, (**D**) 36 hpi, and (**F**) 48 hpi. The network elements were nodes representing molecules and edges representing the interaction between molecules. The node size represents the significance of the term. Upregulated genes associated with each term are shown in red, while downregulated are in green. * *p* < 0.05, ** *p* < 0.01.

Using Cytoscape network analysis software (version 3.9.1), GO terms were clustered into functional groups based on kappa scores (Figure 3A,C,E). GO terms clustered into 7, 16, and 13 functional groups at 24, 36, and 48 hpi, respectively (Figure 3B,D,F). The functional group that was significant across all three time points was a response to type I interferons (IFNs), with 10.0% of all significant GO terms at 24 hpi (Figure 3A,B), 5.88% (Figure 3C,D) and 11.54% (Figure 3E,F) at 36 and 48 hpi. It is compelling to note that the GO terms associated with double-stranded RNA binding (10%; Figure 3A) were involved at 24 hpi, potentially leading to the involvement of the PRRs signaling pathway [30] (23.53%; Figure 3C) at 36 hpi with PHEV. The enrichment of these DEGs early in the infection possibly leads to the involvement of GO terms associated with the adaptive immune response (26.92%), monocyte chemotaxis (11.54%), and motile cilium (11.54%) at 48 hpi (Figure 3E).

### 3.4. Pathway Analysis of DEGs Regulated by PHEV in ALI-PRECs

All significant IPA pathways for each timepoint are provided in (Tables S4–S6). A total of 516, 278, and 388 pathways were significantly enriched (*p* < 0.05) in ALI-PRECs after 24, 36, and 48 hpi with PHEV, respectively. The “Role of Hypercytokinemia and Hyperchemokinemia in the Pathogenesis of Influenza” pathway was significantly enriched with a positive z-score value > 2 at all three time points, thus implying an overall pathway activation (Tables S4–S6). It is beyond the scope of this study to analyze and discuss all the differentially regulated pathways. A pathway-associated analysis with a well-researched influenza virus, also a respiratory virus affecting airway epithelial cells, is ideal for focusing on the current results and discussion.

#### 3.4.1. Early Infection

**Figure 4:**
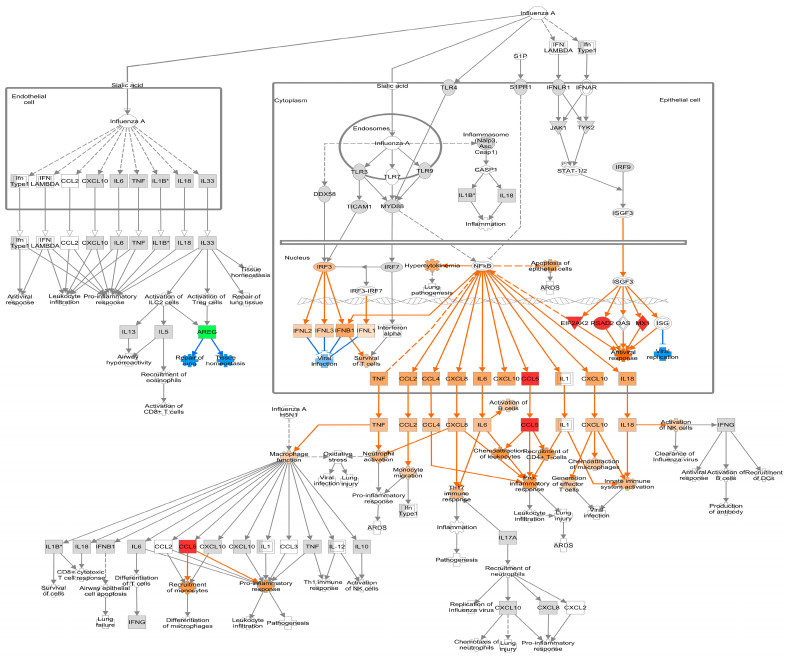
Differential gene expression at 24 h post-inoculation (hpi) within the canonical IPA pathway “Role of Hypercytokinemia/hyperchemokinemia in the Pathogenesis of Influenza”. Downregulated genes are shown with green fill, while upregulated genes were shown with pink fill. The greater the upregulation or downregulation, the darker the fill. A molecule activity predictor tool predicts downstream activity based on significant differential gene expression. Predicted activation is shown in orange, and predicted inhibition is shown in blue. The more confident the prediction, the darker the fill. Solid lines represent direct relationships, while dashed lines represent indirect relationships. * Denotes statistical significance.

Following PHEV infection in ALI-PRECs, at 24 hpi, genes associated with the recognition of viral RNA (*ZNFX1*) [31] and transmembrane proteins associated with transcytosis of immune complexes (*PIGR*) were all upregulated >2 log_2_ fold change (FC) (Table S1). PHEV-induced a robust antiviral response by upregulating several interferon-inducible genes such as *RSAD2*, *CMPK2*, *OAS2*, *OASL*, *MX1*, *MX2*, *PARP14*, *GBP1*, *IFIT1*, *UBE2L6/RIG-B*, *MDA5/IFIH1*, *EIF2AK2/PKR*, and *ISG15* (Figure 4 and Table S1). While most of them were associated with the type 1 interferon response, *RSAD2* induced by type I and type II interferon [32] had >3 log_2_ FC at 24 hpi (Table S1). The early antiviral activity in ALI-PRECs was further evident by the upregulation of *HERC5*, *HERC6*, and zinc finger gene *ZC3HAV1* (Table S1), which inhibit viral replication [33,34,35]. Visualizing these DEGs at 24 hpi, using the “Role of Hypercytokinemia and Hyperchemokinemia in the Pathogenesis of Influenza” pathway, predicts that robust antiviral gene modulation could be through IRF3/IRF7 and other mediators of ISGF3. In addition, the pro-inflammatory response is predicted to be mediated through NFkB signaling (Figure 4).

#### 3.4.2. Mid Infection

**Figure 5:**
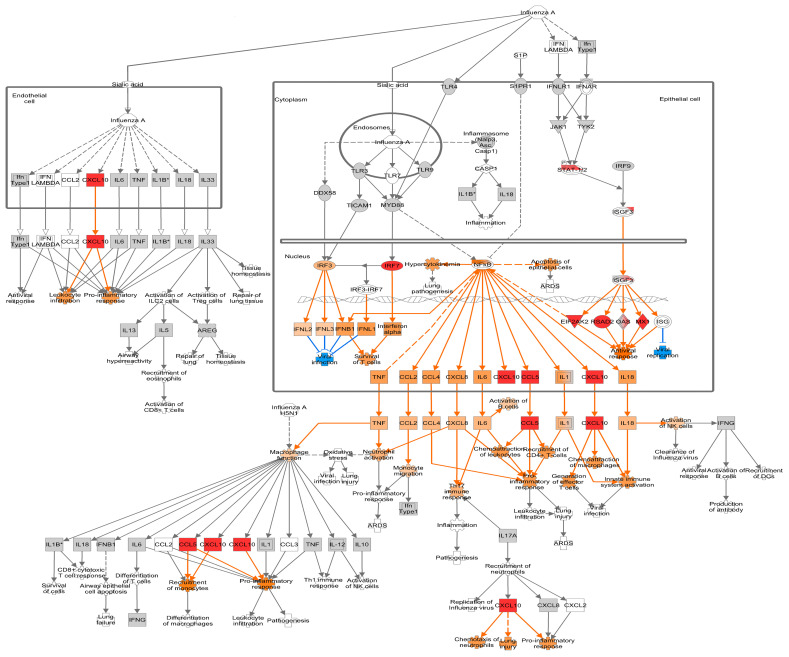
Differential gene expression at 36 h post-inoculation (hpi) within the canonical IPA pathway “Role of Hypercytokinemia/hyperchemokinemia in the Pathogenesis of Influenza”. Downregulated genes are shown with green fill, while upregulated genes are shown with pink fill. The greater the upregulation or downregulation, the darker the fill. A molecule activity predictor tool predicts downstream activity based on significant differential gene expression. Predicted activation is shown in orange, and predicted inhibition is shown in blue. The more confident the prediction, the darker the fill. Solid lines represent direct relationships, while dashed lines represent indirect relationships. * Denotes statistical significance.

Antigen-presenting markers *TNFSF9*, *CD40*, and *CD86* were upregulated by 36 hpi (Table S2). The levels of zinc finger genes associated with antiviral activity, *ZC3HAV1*, *ZC3H12A*, and *ZC3H12C*, were also elevated along with *DEFB1*, an antimicrobial gene. In ALI-PRECs, the antiviral response and inhibitors of viral replication continued at 36 hpi with increased expression of *RSAD2*, *CMPK2*, *OASL*, *OAS2*, *MX1*, *MX2*, *PARP14*, *GBP1*, *EIF2AK2/PKR*, *IFIT1*, *ISG15*, *MDA5/IFIH1*, *UBE2L6/RIG-B*, and *TRIM22*, [36,37] as well as *HERC5*, *HERC6*, and *ZNFX1* (Table S2). The prediction of IRF3/IRF7 role in the transcriptional activation of interferon-stimulated genes (ISG) and antiviral response by IPA analysis at 24 hpi was indeed confirmed by a 1.5-fold change upregulation of *IRF7* at 36 hpi (Figure 5 and Table S2). The strong type I IFN response also increased *STAT1* and *CD274/PD-L1* DEG levels, and their expression is positively correlated [38]. The synergism of IFN response, STAT1 expression, and upregulation of *TNFAIP3*, an NFκB inhibitor, can lead to an induced *CCL5* and *CXCL10*. In addition, the continued upregulation of *CCL5* and *CXCL10* further activates the pro-inflammatory responses, leukocyte infiltration, chemoattraction of macrophages, generation of effector T cells, and innate immune system activation as predicted by IPA analysis (Figure 5). However, the upregulation of *CXCL10* and activation of the pro-inflammatory response can lead to lung injury. Remarkably, one of the most downregulated genes at 36 hpi is *CILK1* (−1.96 log_2_FC; Table S2), which is involved in ciliopathies [39].

#### 3.4.3. Late Infection

**Figure 6:**
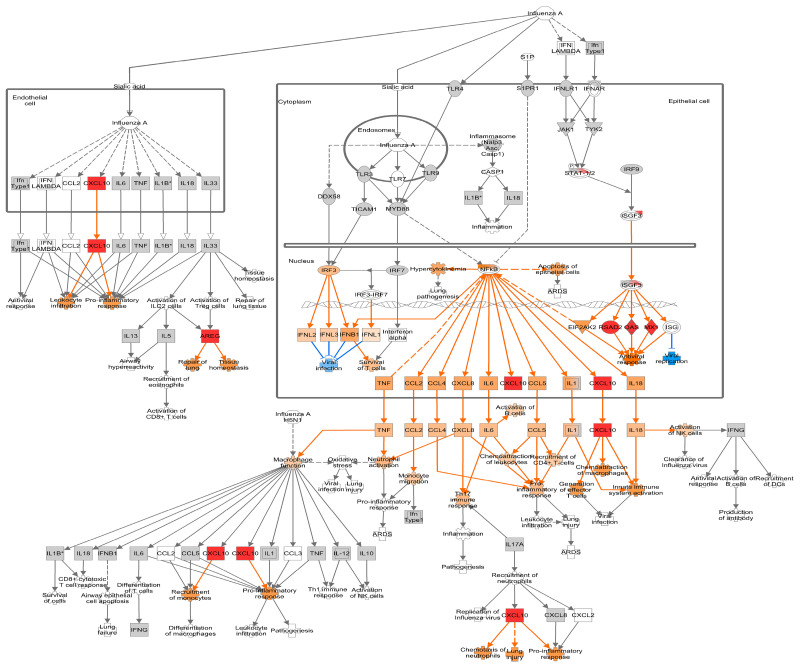
Differential gene expression at 48 h post-inoculation (hpi) within the canonical IPA pathway “Role of Hypercytokinemia/hyperchemokinemia in the Pathogenesis of Influenza”. Downregulated genes are shown with green fill, while upregulated genes are shown with pink fill. The greater the upregulation or downregulation, the darker the fill. A molecule activity predictor tool predicts downstream activity based on significant differential gene expression. Predicted activation is shown in orange, and predicted inhibition is shown in blue. The more confident the prediction, the darker the fill. Solid lines represent direct relationships, while dashed lines represent indirect relationships.

A solid antiviral response continued at 48 hpi (Figure 6 and Table S3). Several interferon stimulatory genes, *TNFAIP3*, *STAT1*, *CXCL10*, and *CXCL11*, were upregulated with predicted pro-inflammatory and chemokine activation. The active inflammatory response was further evident by the downmodulation of regulators of inflammation, *MRC1/CD206*, an inflammation resolution marker, and *MARCO*, a scavenger receptor. All genes (*ROPN1L*, *RSPH1*, *PACRG*, *SPA17*, *DNAH11*, *LRRC23*, *LRRC49*, and *LRRC51*) related to ciliary motility were significantly downregulated < −1 log_2_FC (Table S3). The expression of *CILK1* was significantly increased (1.72 log_2_FC), and *CD164L2*, a sialomucin-like 2 protein, was downregulated (−1.71 log_2_FC) by 48 hpi. Finally, the upregulation of the *AREG* gene was predicted to be involved in the repair of lung and tissue homeostasis, possibly indicating a recovery response.

**Figure 7:**
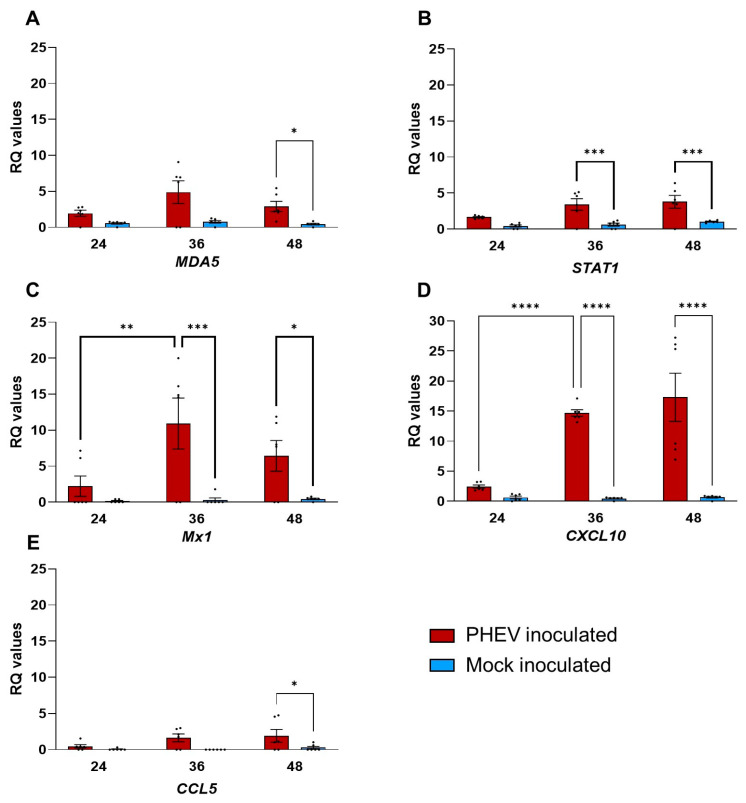
Quantitative PCR analysis of ALI-PRECs inoculated with PHEV or mock inoculated with infection medium. Bar graph showing relative quantification (RQ) levels of *MDA5* (**A**), *STAT1* (**B**), *Mx1* (**C**), *CXCL10* (**D**), and *CCL5* (**E**) measured in ALI-PRECs treated with HA (titer of 128) of PHEV and mock inoculum at respective h post-infection (x axes). RQ values were calculated using the 2−ΔΔCT method. The data are normalized against the geometric mean for three endogenous control genes (*EIF3K*, *PPIA*, and *RPL10*). This graph is generated from three technical replicates and two biological replicates (2 pigs). Statistical analysis was performed using Fisher’s LSD multiple-comparison test (GraphPad Prism 9.0.1). *, *p* value  <  0.05; **, *p* value < 0.001; ***, *p* value < 0.005; ****, *p* value < 0.0001.

The RNA-seq data were further confirmed by normalized qPCR data analysis of *MDA5*, *STAT1*, *Mx1*, *CXCL10*, and *CCL5* genes (Figure 7). The expression of PRR, *MDA5*, was observed as early as 24 hpi and the expression was significant (*p* value  <  0.05) by 48 hpi (Figure 7A). The Jak-STAT signaling molecule *STAT1* gene was significantly (*p* value  <  0.005) upregulated at both 36 and 48 hpi in ALI-PRECs following infection with PHEV (Figure 7B). The interferon stimulatory genes, *Mx1* and chemokine *CXCL10*, were upregulated considerably by 24 hpi and continued into 48 hpi (Figure 7C,D), while upregulation of *CCL5* was significant (*p* value  <  0.05) at 48 hpi (Figure 7E).

## 4. Discussion

The present study further confirms previous work that ALI-PRECs constitute an excellent ex vivo infection model to study early innate immune responses against PHEV [20]. Here, we explored the transcriptome-wide differences during PHEV infection using RNA-seq analysis. The use of ALI-PREC cultures allowed for a time-course analysis of response to PHEV infection, recapitulating a cell culture environment morphologically and functionally more representative of the epithelial lining of the swine trachea than traditional culture systems [14,20]. As in the upper respiratory tract in vivo, PHEV replicated actively in this environment, inducing cytopathic changes and progressive disruption of the mucociliary apparatus systems [14,20]. These changes were also evident at the transcriptomic level throughout the experimental timeline, especially at 48 hpi. For instance, *CILK1*, which is involved in ciliopathies [39], was downregulated at 36 hpi, and eight genes associated with motile cilium, *ROPN1L*, *RSPH1*, *PACRG*, *SPA17*, *DNAH11*, *LRRC23*, *LRRC49*, and *LRRC51* were also downregulated at 48 hpi [40,41,42]. ALI-PRECs were previously shown to produce acidic mucins, and PHEV infection caused a reduction in mucus at 48 hpi [20]. Here, we found that PHEV infection results in a transcriptional decrease of CD164 sialomucin-like 2 proteins (CD164L2) at 48 hpi, further supporting our previous study. CD164 is a type I integral transmembrane sialomucin that functions as an adhesion receptor [43]. Recent studies have identified sialomucin CD164 as an essential viral entry factor for the lymphocytic choriomeningitis virus [44]. However, the specific role of CD164 during PHEV infection remains unknown.

### 4.1. Pattern Recognition Receptors on ALI-PRECs Possibly Initiates a Robust Antiviral Response during PHEV Infection

Respiratory epithelial cells are one of the first cells to react and distinguish pathogens using an array of PRRs, which include TLRs, nucleotide-binding and oligomerization domain (NODs)-like receptors (NLRs), RIG-I-like receptors (RLRs), membrane C-type lectin receptors (CLRs), and DNA receptors, that recognize and distinguish pathogen-associated molecular patterns (PAMPs). Thereby triggering a downstream signaling event leading to the transcriptional upregulation of an inflammatory and antiviral response. In fact, at 36 hpi, the most significant GO functional group by kappa score was the PRR signaling pathway, with 23.53% of significantly enriched GO terms.

At 24 hpi, PHEV triggered mostly PRR molecules associated with the RLR pathway in ALI-PRECs, i.e., *RSAD2*, *ZNFX1*, *OAS2*, *OASL*, *MX1*, *MX2*, *PKR/EIF2AK2*, *IFIT1*, *ISG15*, and *MDA5/IFIH1*. An increase in the expression of *IFIH1* in PHEV-infected ALI-PRECs was also reported previously based on qPCR analysis [20]. ZNFX1 and MDA5 detect viral RNA and initiate signaling cascades [31,45], which has been shown to activate type I IFN and inflammatory cytokine production, even in other porcine coronaviruses [46,47]. During viral infection, MDA5 levels are also enhanced by the activation of PKR [48], which is encoded by the gene *EIF2AK2* [49]. Activation of PKR mediates the activation of NF-kB, apoptosis, and the antiviral response [49]. With the accumulation of viral dsRNA in infected cells, PKR phosphorylation will, in turn, inhibit the translation of viral RNA [50,51], and *PKR* was significantly upregulated at 24 and 36 hpi. A noncanonical sensor for RNA viruses that initiates type 1 IFN response is PARP9, and deletion of PARP9 in human immune cells inhibits type 1 IFN response during reovirus infection [52]. At 36 hpi, the expression of *PARP9* and *DTX3L* increased >1 log_2_FC (Table S2). A hyperactive IFN response can induce PARP9 and DTX3L, binding to form PARP9-DTX3L, an E3 ubiquitin ligase complex, which can promote ISG expression and disrupt viral assembly. PARP9-DTX3L complex with STAT1 can enhance downstream interferon efficacy and can favor host defense more so when there is an increased expression [53]. *STAT1*, a key functional component of the IFN signaling pathway, is upregulated at 36 and 48 hpi by increased *IRF7* expression at 36 hpi. Notably, some of these genes (e.g., *IFN*, *STAT*, *OAS*, *MX*, *IL-8*, and *CXCL10*) were previously reported upregulated (qPCR) in trachea tissues from neonatal piglets infected with PHEV under experimental conditions [14].

The cell surface of ALI-PRECs contain other receptors such as *HERC5*, *HERC6*, *ZNFX1*, *ZC3HAV1*, *ZC3H12A*, *ZC3H12C*, *DEFB1*, *TNFSF9*, *CD40*, and *CD86*, based on current transcriptome data (Tables S1–S3). All these genes were differentially regulated and involved in viral detection, initiation of antiviral response, antigen presentation, transcytosis of immune complexes, inhibition of viral replication, and initiation of the phagocytosis response RNA response [31,33,34,35]. PHEV infection also triggered a response associated with phagocytosis in ALI-PRECs with the modulation of cell surface receptors, *CD274 (PD-L1)*, *B2M* (subunit of MHC-1), *CD24*, *CD206/MRC1* (C-type lectin receptor), and *MARCO* [54,55,56,57,58]. An increase in *CD274*, *B2M* at 36 hpi and ~2log_2_FC increase of *CD24* at 48 hpi (phagocytosis inhibitors), in conjunction with downmodulation (−1.5 log_2_FC) of scavenger receptors *CD206/MRC1* and *MARCO* at 48 hpi is an indication that ALI-PRECs express a “don’t eat me signal” during PHEV infection. In severe human cases of SARS-CoV-2, MARCO-positive alveolar macrophages were depleted [59], which may be a consequence of betacoronavirus infection. APOBEC1 is shown to edit mRNA transcripts, leading to altered cellular function [60]. Similarly, APOBEC1 can cause host-driven editing of viral RNA, including SARS-CoV-2, leading to C-to-U substitution mutations that can impact viral replication and viral progeny production [61,62]. In ALI-PRECs infected with PHEV, *APOBEC1* upregulated (>1.0 log_2_FC) at 36 and 48 hpi, leading to possible mutations in PHEV RNA, but further studies are warranted to confirm the above findings.

### 4.2. Interferon Response in ALI-PRECs and the Role of RSAD2 following PHEV Infection

During infection, i.e., 24 to 48 hpi, PHEV significantly enhanced the number of DEGs in ALI-PRECs with expression mostly belonging to IFN functional groups. Several ISGs were differentially expressed in this study, including *OAS2*, *OASL*, *MX1*, *MX2*, *ISG15*, *IFIT1*, *PARP14*, *GBP1*, *RIG-B*, *TRIM22*, *CMPK2*, and *RSAD2*, as shown in Figure 4, Figure 5 and Figure 6 and Tables S1–S3.

PHEV PAMPs, upon binding to the PRRs, activate ISGs such as *OAS2* (also known as *2*′-*5*′*OAS*, *2*′-*5-oligoadenylate synthase 2*), which plays a vital role in the interferon-mediated antiviral pathway [63]. The porcine OAS family consists of three functional genes (OAS1a, OAS1b, and OAS2) encoding active enzymes to catalyze adenosine triphosphate in 2′-specific nucleotidyl transfer reactions to synthesize 2′,5′-oligoadenylates (2–5 As) [63,64]. Another OAS homolog, OASL, has dual functions that depend on the phase of viral infection and various mechanisms and is postulated to interfere with the 2–5 A and RNase L pathway [65]. The OAS family members activate latent RNase L, and antiviral activity mediates through the OAS/RNase L pathway, an interferon effector pathway that induces viral RNA degradation and inhibits viral replication [66]. Both in ALI-PRECs and porcine tracheal tissue, a significant increase in the expression of *OAS1* in response to PHEV infection has previously been reported [20]. Similarly, in the current study, a significant upregulation (≥2 log_2_FC) of *OAS2* and *OASL* was consistent at all three time points, while *RNASEL* was upregulated (>1 log_2_FC) alongside OAS genes at 36 hpi. Recent findings in multisystem inflammatory syndrome in children with SARS-CoV-2 demonstrate that genetic deficiencies of OAS1, OAS2, or RNase L displayed exaggerated inflammatory responses [67]. It will be interesting to evaluate the role of the OAS/RNase L pathway in mediating the cytokine response during PHEV infection.

Interferon-induced Mx GTPases, *MX1* and *MX2*, were also significantly upregulated (>1.5 log_2_FC) at all three time points. The crucial role of MX1 in influenza resistance has been well documented in mouse and human studies, where it was shown to prevent the transcription and replication of viruses that utilize nuclear replication by preventing the transcription of viral RNA polymerase [68]. Again, *MX1* upregulation was also reported in PHEV infected ALI-PRECs and porcine tracheal tissues [20]. Although the exact role of *MX1* in inhibiting PHEV is not yet fully understood, higher levels of expression of *MX1* in the blood have been associated with a reduced risk of developing severe disease in SARS-CoV-2 infections [69]. While not as well studied as *MX1*, *IFIT1* encodes for a family of IFN-induced proteins with tetratricopeptide repeats that are induced after viral infection or PAMP recognition [70]. These proteins inhibit the replication of multiple families of viruses through distinct mechanisms, including translation inhibition, recognizing a lack of 2′-O methylation, 5′-ppp RNA recognition, and binding to viral proteins [70].

*ISG15* gene is critical in host antiviral response with diverse and pathogen-dependent mechanisms to protect the host during infection [71] by conjugating to a wide range of viral and cellular proteins [72]. Many viruses that encode viral proteases have evolved mechanisms to reverse ISG15 conjugation from viral target proteins, including the betacoronaviruses SARS-CoV and MERS-CoV [71,72]. ISG15 can be associated with HERC5, IFIT1, PKR, and TRIM25 and enhance antiviral signaling by prolonging the state of signaling proteins such as IRF3 and STAT1 or can inhibit activation state by reversing the ISGylation via USP18 [72,73]. PHEV significantly upregulated ISG15 expression (>1.5 log_2_FC) at all three time points, and it appears ISG15 does interact with *HERC5*, *IFIT1*, *PKR*, *TRIM25*, *STAT1*, and *USP18*, as their expression was also enhanced (>1.0 log_2_FC) at various timepoints during ALI-PRECs infection (Tables S1–S3). However, the interactions of ISG15 with various cellular and viral proteins during PHEV infection. An enhanced expression of pattern recognition receptors such as *TRIM25*, *TRIM38*, *MDA5/IFIH1*, and *DDX58* can also increase *IRF7*, a regulator of type 1 IFN response [36,74,75,76].

The PAMPs of PHEV also activated *RSAD2* or *viperin* and *CMPK2* in ALI-PRECs at all three time points. In humans, both RSAD2 and CMPK2 are adjacent to each other in the short arm of chromosome 2, and both are induced by IFN signaling (reviewed by [77]). RSAD2 aids in the production of 3′-deoxy-3′, 4′-dihydro-CTP (ddhCTP) that causes premature termination of RNA synthesis by acting as a chain terminator for the viral RNA-dependent RNA polymerase (RdRP) of some viruses [77]. CMPK2 has been implicated as an antiviral gene with activity against multiple coronaviruses [78]. A co-expression of CMPK2 along with RSAD2 further enhances the ddhCTP production, and it is speculated that they may play a role in inflammation. *RSAD2* is highly conserved in mammalian evolution, and a number of overexpression and knockdown experiments have demonstrated its importance in antiviral activity [77,79], including members of the *Coronaviridae* family [80]. For example, RSAD2 has been shown to inhibit replication of the coronavirus porcine epidemic diarrhea virus (PEDV), which is believed to be a result of the interaction of the RSAD2 S-adenosylmethionine domain with the N protein of PEDV [81]. While the role of host cell antiviral responses is comparatively well characterized in other porcine coronaviruses [82,83,84], additional studies are required to delineate the antiviral responses in PHEV infection.

### 4.3. Chemokine Response

In addition to inhibiting viral replication by the interferon response, ALI-PRECs strongly expressed the chemokines *CCL5/RANTES* and *CXCL10/IP-10* following PHEV infection. Both of these chemokines were previously reported to be upregulated in PHEV-infected ALI-PRECs by qPCR studies [20]. In the present study, *CCL5* was significantly upregulated at 24 and 36 hpi, and *CXCL10* was significantly upregulated at 36 and 48 hpi, as shown in Figure 4, Figure 5 and Figure 6. Activation of chemokines is a classic response during infection, which mediates immune cell trafficking with potential impacts on pathogenesis, virus replication, virus clearance, and healing. Low levels of CCL5 and a high SARS-CoV-2 viral load were associated with the severity of COVID-19 disease [85]. Virus-infected cell clearance is impaired in influenza infected mice lacking CCL5 or by blocking CCL5 during respiratory syncytial virus infection, causing further tissue damage and a pro-inflammatory response [86,87]. Similarly, in mice infected with the betacoronavirus murine hepatitis virus (MHV), the absence of CXCL10 is associated with increased virus titers and high mortality [88]. In addition, CXCL10 was demonstrated to limit the spread of MHV infection [89]. Both CCL5 and CXCL10 were reported to be predictive biomarkers for clinical outcomes of COVID-19, and it appears this observation can be broadly applied to other betacoronaviruses, including PHEV.

## 5. Conclusions

This is the first study to apply transcriptomic analysis to organotypic cultures of porcine respiratory epithelial cells over the course of infection with the betacoronavirus PHEV. It contributes to understanding the molecular mechanisms driving the early antiviral innate immune response in the respiratory tract with a robust mucociliary response. At this primary site of infection, the overall significant responsive pathways that were activated leading toward an effective response to PHEV include IFN-, PRR-, and chemokine-mediated pathways. Moreover, the results presented herein highlight the vital role of respiratory epithelial cells in maintaining respiratory homeostasis and the initiation, resolution, and outcome of the infection process. Finally, this study further demonstrates the potential of the organotypic respiratory culture system as a robust infection model alternative or as a complement to live animals, which can be readily applied across animal species and pathogens.

## Data Availability

Data are contained within the article or Supplementary Materials. Additional raw data formatted according to the Gene Expression Omnibus (GEO) requirements are available in the NCBI SRA database under BioProject ID PRJNA1029154.l.

## Supplementary Materials

The following supporting information can be downloaded at: https://www.mdpi.com/article/10.3390/v16060939/s1, Table S1: 24 hpi DEG; Table S2: 36 hpi DEG; Table S3: 48 hpi DEG; Table S4: 24 hpi Pathways; Table S5: 36 hpi Pathways; Table S6: 48 hpi Pathways.
